# Influence of HTLV-1 in the clinic, microbiologic and immunologic features of tuberculosis

**DOI:** 10.1186/1742-4690-8-S1-A45

**Published:** 2011-06-06

**Authors:** Maria de Lourdes S Bastos, Brooke Finkmoore, Silvane Santos, Ohana Bispo, Tasso Barreto, Ingrid Cardoso, Iana Bispo, Flávia Bastos, Lee Riley, Edgar M Carvalho

**Affiliations:** 1 Serviço de Imunologia, Complexo Hospitalar Universitário Professor Edgard Santos, Universidade Federal da Bahia, Salvador-BA, Brazil; 2 Escola Bahiana de Medicina e Saúde Pública, Salvador-BA, Brazil; 3 Hospital Especializado Octávio Mangabeira, Salvador-BA, Brazil; 4 University of California at Berkeley, USA; 5 Instituto Nacional de Ciência e Tecnologia de Doenças Tropicais (CNPq/MCT), Salvador-BA, Brazil

## Background

HTLV-1 infection increases susceptibility and severity of tuberculosis. Despite the documentation of a decreased in vitro and in vivo T cells response to mycobacterial antigens, the reasons of the HTLV-1 infection increase the susceptibility and make worse the clinic course of tuberculosis are not understood. For instance, it is not known if HTLV-1 infection increases bacillary load and if severity of tuberculosis is due to the inability of these patients to control bacterial growth. The aim of the present study was to evaluate how HTLV-1 might influence the clinic, bacteriologic and immunologic features of tuberculosis.

## Material and methods

A prospective cohort studies enrolled 13 cases of tuberculosis associated with HTLV-1 (cases) and 25 patients with tuberculosis without HTLV-1 (controls). Clinical findings, bacillary load in the sputum, x-rays, immunological response and death were compared in the two groups.

## Results

There was no difference in the demographic features, clinical manifestations and in vivo response to PPD. IFN-γ were higher in unstimulated cultures of mononuclear cells of HTLV-1 patients with tuberculosis than in controls (P<0.01). The enhancement in TNF-α and IFN-γ responses after stimulation with PPD was similar in cases and controls. There was no difference in the bacillary load among the groups but baciloscopy became negative faster in cases than in controls. Death only occurred in two co-infected patients.

## Conclusion

Severity of tuberculosis in patients co-infected with tuberculosis may be due to the enhancement in the Th1 inflammatory response, rather than a decreased ability to control bacteria growth.

